# Prevalence of endotoxemia after surgery and its association with ICU length of stay

**DOI:** 10.1186/cc7934

**Published:** 2009-06-29

**Authors:** Franco Valenza, Lorella Fagnani, Silvia Coppola, Sara Froio, Francesca Sacconi, Cecilia Tedesco, Micol Maffioletti, Marta Pizzocri, Valentina Salice, Maria Luisa Ranzi, Cristina Marenghi, Luciano Gattinoni

**Affiliations:** 1 Dipartimento di Anestesia, Rianimazione (Intensiva e Subintensiva) e Terapia del Dolore, Fondazione IRCCS – "Ospedale Maggiore Policlinico Mangiagalli Regina Elena", Via Francesco Sforza 35, 20122, Milano, Italy; 2 Dipartimento di Anestesiologia Terapia Intensiva e Scienze Dermatologiche, Università degli Studi di Milano, Via Festa del Perdono 7, 20122, Milano, Italy; 3 Laboratorio Centrale di Analisi Chimico Cliniche e Microbiologiche; Fondazione IRCCS – "Ospedale Maggiore Policlinico Mangiagalli Regina Elena", Via Francesco. Sforza 35, 20122, Milano, Italy

## Abstract

**Introduction:**

The aim of this observational study was to investigate the prevalence of endotoxemia after surgery and its association with ICU length of stay.

**Methods:**

102 patients admitted to a university ICU after surgery were recruited. Within four hours of admission, functional data were collected and APACHE II severity score calculated. Arterial blood samples were taken and endotoxemia was measured by chemiluminescence (Endotoxin Activity (EA)). Patients were stratified according to their endotoxin levels (low, intermediate and high) and according to their surgical procedures. Differences between endotoxin levels were assessed by ANOVA, accepting *P *< 0.05 as significant. Data are expressed as mean ± SD.

**Results:**

EA levels were low in 68 (66%) patients, intermediate in 17 (17%) and high in 17 (17%). Age (61 ± 17 years) and APACHE II score 8.3 ± 3.7 (*P *= 0.542) were not significantly different in the three EA groups. Functional parameters on admission were similar between EA groups: white blood cells 11093 ± 4605 cells/mm^3 ^(*P *= 0.385), heart rate 76 ± 16 bpm (*P *= 0.898), mean arterial pressure 88.8 ± 13.6 mmHg (*P *= 0.576), lactate 1.18 ± 0.77 mmol/L (*P *= 0.370), PaO_2_/FiO_2 _383 ± 109 mmHg (*P *= 0.474). Patients with high levels of EA were characterized by longer length of stay in the ICU: 1.9 ± 3.0 days in the low EA group, 1.8 ± 1.4 days in intermediate and 5.2 ± 7.8 days in high group (*P *= 0.038).

**Conclusions:**

17% of our patients were characterized by high levels of endotoxemia as assessed by EA assay, despite their low level of complexity on admission. High levels of endotoxin were associated with a longer ICU length of stay.

## Introduction

Endotoxin is a constituent of the cell wall of Gram-negative bacteria capable of inducing potent inflammatory response in the host [1,2]. Isolated and purified from the wall of several Gram-negative bacteria, it has been used to investigate many aspects of the immuno-inflammatory response of sepsis through inoculation in the laboratory animal [3,4] or in humans [5-7]. However, endotoxin has also been documented in clinical scenarios such as trauma or burn injury [8-10]. Rather than a manifestation of exogenous infection, translocation of lipopolysaccharide across the intestinal membrane when permeability is increased is the putative mechanism of these forms of endotoxemia [11,12].

Patients undergoing surgery may have direct shedding of lipopolysaccharide into the circulation via manipulation of infected surgical sites, violation of natural barriers such as bowel resection, contamination from the environment, or via the use of invasive devices. Meanwhile, postural changes, blood loss, and vasoplegia all cause relative or absolute hypoperfusion that may favor bacterial translocation. Buttenschoen and colleagues have in fact shown that major abdominal surgery is associated with transient endotoxemia [13,14]. However, while the likelihood of endotoxemia might be straightforward in major abdominal surgery, less is known in other surgical procedures, apart from cardiopulmonary bypass [15,16].

We studied the prevalence of endotoxemia in a population of patients admitted to an intensive care unit (ICU) after surgery and the relation between endotoxemia and their outcome.

## Materials and methods

The study was approved by our institution ethics committee, and informed consent was obtained from all patients. Adult patients admitted to the ICU of our institution were recruited for the study unless they were transferred from another ICU, had no arterial line in place, or were on chronic dialysis.

On admission and the next morning, clinical history and laboratory data were taken. Cardio-respiratory variables were recorded and Acute Physiology and Chronic Health Evaluation (APACHE) II score was calculated [17]. Systemic Inflammatory Reaction Syndrome (SIRS) was considered to be present when at least two of these criteria were met: temperature above 38°C or below 36°C, heart rate of more than 90 beats/min, respiratory rate of more than 20 breaths/min or partial pressure of carbon dioxide of less than 32 mmHg, or white blood cell count above 12,000 mm^3 ^or below 4000 mm^3 ^[18]. Within four hours after admission blood was withdrawn for Endotoxin Activity (EA) assay. During the course of the ICU stay blood and other biologic specimens were collected on a clinical basis and sent to the microbiologic laboratory of the institution for microorganism detection. Length of stay and mortality of both ICU and hospital were calculated. Clinicians were unaware of the results of the EA assay throughout patient's ICU and hospital stay.

### Endotoxin activity assay

The EA assay has been described in detail previously [19]. Briefly, the method allows the measurement of EA as a function of each patient's neutrophil chemiluminescence's activity (on a scale from 0 to 1). An EA level of 0.4 is approximately equivalent to an endotoxin concentration of 25 to 50 pg/mL, and a level of 0.6 equivalent to 100 to 200 pg/mL. A 2 ml sample of whole blood was drawn through an indwelling arterial line into an endotoxin-free blood collection tube (Vacutainer systems; Becton Dickinson, Franklin Lakes, NJ, USA). Blood samples were maintained at room temperature and assayed within 30 minutes of collection. To assay levels of endotoxin, a 10 μl aliquot of whole blood was placed in each of three tubes containing luminol buffer (300 μl/tube). The control tube contained blood and buffer only, whereas a positive control contained a maximum stimulatory concentration of endotoxin (2 ng/ml); the final tube contained the test sample. All three tubes were incubated at 37°C for five minutes and assayed in duplicate. Chemiluminescence was initiated by the addition of 20 μl/tube human complement opsonized zymosan. Continuous measurements were made of light emissions at 30-second intervals over a total period of 20 minutes in a reciprocating tube luminometer (Autolumat LB 953; E. G. & G. Berthold, Wildbad, Germany). Quality control assays were performed on a 1:1 ratio basis (i.e. one control every sample measured). Based on the results obtained, samples were considered adequate if the coefficient of variation between duplicates was lower than 15% if EA level was below 0.2 and 30% if it was above 0.2.

### Statistical analysis

A descriptive analysis was first conducted on the entire population. Patients were then stratified according to the EA results into the following groups: low (EA <0.4), intermediate (EA 0.4 to <0.6) and high (EA ≥ 0.6). To compare continuous variables on admission such as age, APACHE II score, and functional parameters based on both surgical or EA stratification, one-way analysis of variance was used and all pairwise multiple comparisons were assessed by Student Newman-Keuls test. If the normality test (Kolmogorov-Smirnov) failed, data were analysed by Kruskal-Wallis one-way analysis of variance and for all pairwise multiple comparisons procedures Dunn's method was used. Functional parameters at entry and on the next morning were analyzed according to EA stratification by means of two-way analysis of variance. This was also used to assess the interaction between EA levels and the type of surgery in determining ICU and hospital length of stay. For statistical purposes, patients were stratified according to the different kind of surgical procedures they underwent into major surgery and other procedures. Data are presented as mean ± standard deviation, unless otherwise specified. Statistical significance was accepted as *P *< 0.05. The Sigma Stat for Windows version 3.11 (Systat Software Inc, Pont Richmond, CA, USA) was used.

## Results

A total of 122 patients were recruited for the study. All patients had their EA level measured on admission. However, 20 patients were excluded from analysis: 17 because variation of coefficients were out of the accepted range, two because the calculation of EA level was unreliable (value > 1), and one because quality control failed. Therefore, a total of 102 patients were considered.

Out of the 102 patients included in the study, 27 underwent thoracic surgical procedures including 25 resective procedures and 2 decortications; 27 patients underwent abdominal procedures including gastric, intestine, and colon rectum surgery; 20 obese patients underwent gastric banding; 11 patients underwent hepatectomies; 7 underwent urological proceures including 3 procedures on the bladder, 3 prostatectomies, and 1 nephroureterectomy. The remaining 10 patients underwent procedures other then those above mentioned including three timectomies, three femoral bone fracture repair, two median abdominal wall laparocele synthesis, and two tiroidectomies. Patients were scheduled for elective post-operative ICU monitoring. Five patients were admitted because of intra-operative complications. Patients demographics were similar between surgical groups except for obese patients who underwent gastric banding that were younger (*P *< 0.05) and had lower APACHE II scores (*P *< 0.05). Data are shown on Table 1.

**Table 1:** Characteristics of the study population

	**All**	**Thoracic**	**Abdominal**	**Obesity**	**Hepatic**	**Urological**	**Other**
N. of patients	102	27	27	20	11	7	10
Age (years)	62 ± 17	67 ± 8	69 ± 17	40 ± 9	58 ± 18	64 ± 14	69 ± 17
Surgery (min)	178 ± 95	166 ± 64	200 ± 111	121 ± 38	267 ± 106	297 ± 80	146 ± 103
Unscheduled (n)	5	-	2	-	-	-	3
APACHE II score	8.4 ± 4	9.1 ± 3	9.7 ± 4	4.7 ± 2	8.4 ± 5	9.0 ± 3	9.3 ± 4
EA activity	0,3 ± 0.3	0.4 ± 0.2	0.3 ± 0.2	0.4 ± 0.2	0.3 ± 0.3	0.3 ± 0.3	0.4 ± 0.1
LOS ICU (days)	1 (1 to 2)	1 (1 to 2)	1 (1 to 4.7)	1 (1 to 1)	1 (1 to 1)	1 (1 to 1)	1 (1 to 2)
Mortality ICU (n)	3	-	2	-	-	-	-
LOS hosp (days)	8 (5–11)	8.5 (6–13)	10 (7–16)	4 (4–5)	8.0 (6–10)	12 (9–13)	6.5 (5–10)
Mortality hosp (n)	1	1	-	-	-	-	-

Characteristics of the study population are shown. Intensive care unit (ICU) and hospital length of stay (LOS) are presented as median and interquartile ranges. Other data are presented as mean ± standard deviation.

APACHE = Acute Physiology and Chronic Health Evaluation; EA = endotoxin activity.

**Figure 1:**
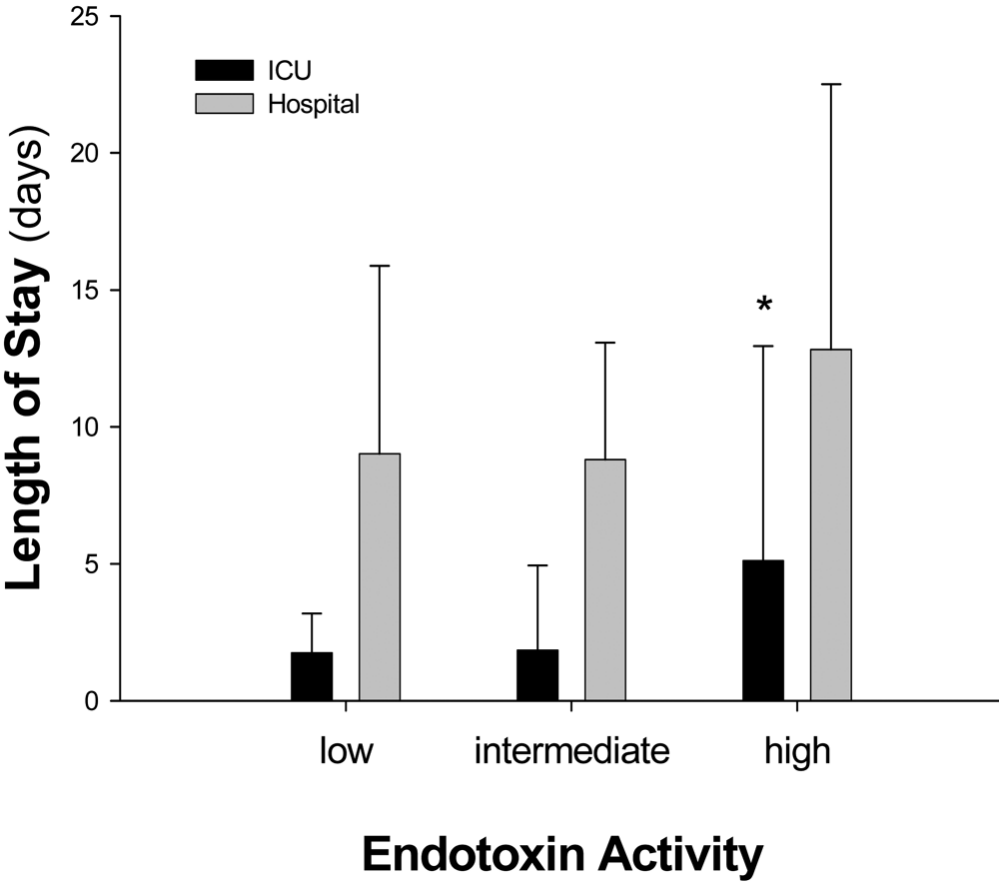
Intensive care unit and hospital length of stay according to endotoxin activity stratification. Intensive care unit (ICU) = black columns; Hospital = gray columns.

**Figure 2:**
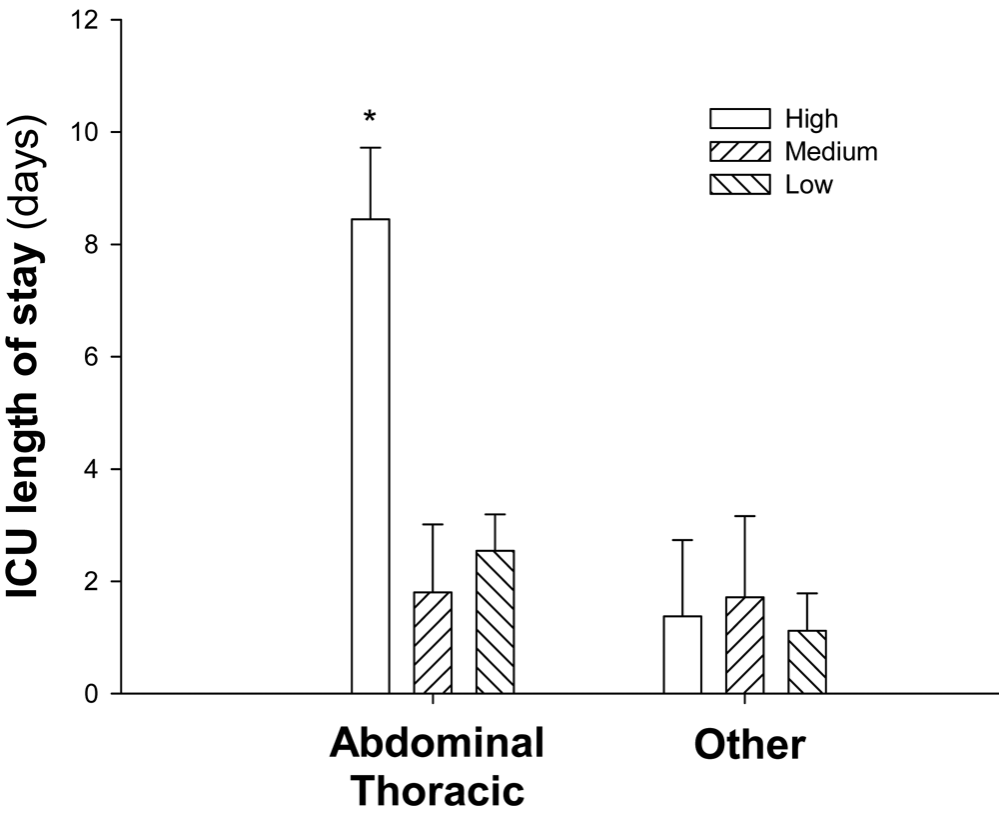
Intensive care unit length of stay according to endotoxin activity stratification within surgical stratification. White columns represent data from patients with high endotoxin activity (EA) levels, while dashed columns refer to patients with intermediate or low EA levels.

Median EA level was 0.282 (25% 0.190, 75% 0.462). When stratified according to EA levels, 68 (66%) had normal values, 17 (17%) had intermediate, and 17 (17%) had high levels. As shown in Figure 1, patients with high EA levels (>0.6) on admission were characterized by a longer ICU length of stay (*P *= 0.038); in hospital length of stay was not different between groups (*P *= 0.387). In patients with high EA activity functional parameters were similar on admission to the other EA stratification groups except for a somewhat higher temperature. However, in contrast to patients without high EA levels, the number of SIRS criteria, white blood cell count, and lactate levels did not improve over time from the first day. Data are shown on Table 2. Patients who underwent major surgery (abdominal or thoracic procedures) and presented to the ICU with higher EA levels were characterized by significantly longer ICU length of stay (Figure 2). These patients were characterized intraoperatively by a slightly worse oxygenation despite a more aggressive ventilatory management; the hemodynamic status was similar between EA stratification groups. Data are shown on Table 3. Complications that resulted in longer ICU stay included: respiratory insufficiency (n = 11), septic shock (n = 2), caridac arrhythmia (n = 3), and pneumonia (n = 1). A total of 152 specimens from 41 patients were sent to the microbiologic laboratory during the ICU stay. Of these, 46 were positive in a total of 13 patients. The characteristics of these patients are summarised in Table 4.

**Table 2:** Ventilator settings and functional parameters on admission to the intensive care unit

		**Low**	**Intermediate**	**High**
Ventilated patients	Admission	62/68 (91%)	16/17 (94%)	17/17 (100%)
	Day 1	6/68 (9%)*	1/17 (6%)*	1/17 (6%)*
Tidal volume (l/min)	Admission	6.5 ± 1.8	6.4 ± 1.2	7.3 ± 1.9
	Day 1	/	/	/
Respiratory rate (atti/minuto)	Admission	11 ± 4	11 ± 2	12 ± 7
	Day 1	18 ± 5*	19 ± 5*	16 ± 4*
PaO_2_/FiO_2 _(mmHg)	Admission	379 ± 111	413 ± 113	372 ± 96
	Day 1	329 ± 62*	330 ± 46*	299 ± 83*
PaCO_2 _(mmHg)	Admission	38.6 ± 4.9	35.1 ± 5.6^#^	37.7 ± 5.0
	Day 1	39.9 ± 4.7	39.3 ± 5.1*	38.8 ± 4.1
pH arterial blood	Admission	7.41 ± 0.05	7.42 ± 0.06	7.39 ± 0.06
	Day 1	7.43 ± 0.03	7.43 ± 0.03	7.43 ± 0.04
Lactate (mmol/l)	Admission	1.12 ± 0.57	1.23 ± 0.47	1.40 ± 1.39
	Day 1	0.79 ± 0.38*	0.81 ± 0.37*	0.89 ± 0.57
Heart rate (bpm)	Admission	76 ± 16	75 ± 17	77 ± 18
	Day 1	82 ± 15*	80 ± 12	84 ± 15
Mean arterial pressure (mmHg)	Admission	88 ± 13	87 ± 13	92 ± 17
	Day 1	91 ± 12	94 ± 13	93 ± 16
Central venous pressure (mmHg)	Admission	7.5 ± 2.5	6.3 ± 2.9	7.5 ± 2.2
	Day 1	6.1 ± 2.6	6.1 ± 2.5	6.1 ± 2.7
Hemoglobin (g/dl)	Admission	12.0 ± 3.5	12.4 ± 1.8	12.4 ± 1.6
	Day 1	11.3 ± 1.6	11.5 ± 1.7	12.0 ± 1.3
Creatinine (mg/dl)	Admission	0.99 ± 0.92	1.06 ± 0.68	0.89 ± 0.37
	Day 1	1.04 ± 1.12	1.05 ± 0.47	0.85 ± 0.27
Azotemia (mg/dl)	Admission	36 ± 24	40 ± 21	37 ± 21
	Day 1	37 ± 27	41 ± 24	36 ± 25
Glycemia (mg/dl)	Admission	134 ± 30	134 ± 25	132 ± 35
	Day 1	110 ± 29*	111 ± 23*	105 ± 21*
Sodium (Na^+^; mEq/l)	Admission	137.5 ± 2.5	137.1 ± 2.2	137.8 ± 2.1
	Day 1	137.9 ± 2.7	138.4 ± 3.5	138.1 ± 2.7
Potassium (K^+^;mEq/l)	Admission	3.9 ± 0.5	3.9 ± 0.5	3.9 ± 0.4
	Day 1	4.1 ± 0.3*	4.0 ± 0.4*	3.9 ± 0.4
Temperature (°C)	Admission	35.2 ± 0.9	34.5 ± 0.9	35.4 ± 1.2^#^
	Day 1	36.6 ± 0.6	36.7 ± 0.5	36.8 ± 0.5
WBC (10^3^/mm^3^)	Admission	10.8 ± 4.2	12.5 ± 5.5	10.8 ± 5.1
	Day 1	9.4 ± 3.0*	10.4 ± 4.0	10.2 ± 2.7
SIRS criteria	Admission	1.4 ± 0.7	1.8 ± 0.8	1.4 ± 0.9
	Day 1	0.9 ± 0.9*	1.3 ± 1.1*	0.9 ± 0.8

Ventilatory and functional parameters collected within four hours of admission to the intensive care unit (Admission) and the morning after (Day 1) are shown in the table. Patients were stratified according to their endotoxin activity levels on admission. * *P *< 0.05 Admission vs Day 1; # p < 0.05 between EA stratification groups. FiO_2 _= fraction of inspired yoghurt; PaCO_2 _= partial pressure of arterial carbon dioxide; PaO_2 _= partial pressure of arterial oxygen; SIRS = Systemic Inflammatory Reaction Syndrome; WBC = white blood cell.

**Table 3:** Intra-operative variables of the patients who underwent major surgery

	**Low**	**Intermediate**	**High**
Number of patients (n)	34	10	10
Age (years)	69 ± 15	67 ± 8	67 ± 12

Duration of surgery (min)	174 ± 101	183 ± 73	189 ± 86

Arterial pressure – OR (mmHg)	121 ± 11	118 ± 13	123 ± 11
Arterial pressure – Preop (mmHg)	136 ± 17	140 ± 14	135 ± 11
pH	7.388 ± 0.05	7.420 ± 0.05	7.392 ± 0.04
BE	-0.9 ± 2.5	-0.6 ± 1.9	-0.4 ± 2.4
Urine output (ml/h)	239 ± 193	331 ± 346	181 ± 98
Cristalloids (mL)	3045 ± 1590	2895 ± 917	3078 ± 900
Colloids (mL)	580 ± 280	533 ± 57	500 ± 0
Blood transfusion (n)	5	1	1

PaO_2_/FiO_2 _(mmHg)	307 ± 141	275 ± 193	243 ± 145
Tidal volume (mL)	679 ± 102	667 ± 129	715 ± 109
Respiratory rate (bpm)	9.9 ± 1.5	9.8 ± 0.6	9.9 ± 1.1
Peak airway pressure (cmH_2_O)	23.8 ± 6.3	20.8 ± 5.9	24.3 ± 6.2
PEEP (cmH_2_O)	2.4 ± 2.3	0.9 ± 2.1	3.5 ± 2.4 *
Invasiveness (n)	5.1 ± 1.1	5.4 ± 0.8	5.4 ± 0.8
Contaminated surgery (n)	2	-	-

Intra-operative variables of the patients who underwent abdominal or thoracic surgery are presented. * *P *< 0.05 analysis of variance. Arterial pressure – OR = mean systolic arterial pressure recorded during the intervention; Arterial pressure – Preop = systolic arterial pressure taken at the time of the preoperative evaluation; BE = base excess; FiO_2 _= fraction of inspired yoghurt; PaO_2 _= partial pressure of arterial oxygen; PEEP = positive end-expiratory pressure. Invasiveness = sum of invasive procedure including tracheal tube, periferal vein, central vein, arterial line, nasogastric tube, bladder catheter.

**Table 4:** Characteristics of the 13 patients who were positive for microbiologic investigations

EA level	Age	Surgery	APACHE	SIRS	Gram +	Gram -	Other	Time	ICU	Hosp	Alive
0.140	77	A	11	2		Klebsiella		Late	4	20	Y
0.446	50	T	6	2			Candida	Late	1	6	Y
0.432	73	O	13	2		Contaminants		Late	2	6	Y
0.125	78	A	9	2		Pseudomonas		Late	24	26	Y
0.333	71	A	9	1	S. epidermidis			Early	1	12	Y
0.235	43	A	10	2	S. aureus			Early	2	12	Y
0.330	90	O	11	2		Enterobacter		Early	1	32	Y
0.191	72	A	10	2	S. aureus			Early	8	19	Y

0.558	59	T	4	2		Pseudomonas		Early	5	9	Y
0.498	36	O	3	3	Enterococcus			Early	5	19	Y

0.750	78	A	7	1		Pseudomonas		Early	28	-	N
0.825	67	A	13	3		Morganella M		Late	22	-	N
0.740	76	T	8	1		Klebsiella		Late	7	29	Y

Table includes endotoxin activity (EA) level, age, type of surgery (A = abdominal, T = thoracic, O = other), Acute Physiology and Chronic Health Evaluation (APACHE) score, numbers of Systemic Inflammatory Reaction Syndrome (SIRS) criteria met at the time of intensive care unit (ICU) admission, strain of microrganisms isolated, whether the microbiological samples were collected early after the admission (E – within three days) or at a later time (L); the ICU and hospital length of stay, survival.

## Discussion

This observational study investigates the prevalence of endotoxemia in a population of patients admitted to the ICU after surgery and evaluates the association between endotoxin levels and outcome. We found that 17% of the patients had levels of endotoxin higher than normal on admission despite the low level of complexity, and that patients with high endotoxin levels had longer ICU length of stay.

To detect endotoxemia we chose to use the EA assay as opposed to the more classic limulus-amebocyte-lysate (LAL) test [20,21] This method is based on the detection of enhanced respiratory burst activity in neutrophils following their priming by complexes of endotoxin and a specific anti-endotoxin antibody [19]. The method allows the expression of EA as a function of each patient's neutrophil chemiluminescence's activity (on a scale from 0 to 1). The technique has been validated against the LAL test [22], and has been recently used in a multicenter trial to assess endotoxin prevalence in a mixed surgical/medical ICU cohort of patients recruited across North America and Europe [23]. According to this method, most of the patients admitted to the ICU after surgery had normal levels of endotoxin. However, 17 out of 102 had higher than normal levels of endotoxin despite their low level of complexity (APACHE score) These data confirm previous observations that circulating endotoxin is a common finding in ICU patients [23], but add to the knowledge on endotoxin prevalence in post-operative patients. In fact, while endotoxemia in patients undergoing major abdominal procedures has been previously shown [13,14], our observation extend to other kind of surgical interventions less likely to be characterized by endotoxemia.

Subjects with high EA levels had a longer ICU length of stay and a trend towards longer hospital length of stay (Figure 1). Interestingly, functional parameters on admission were almost normal and similar between groups of patients stratified by EA levels (Table 2). Subjects with high EA on admission, despite being similar to the other groups with respect to functional data, demonstrated that white blood cell count, SIRS criteria, and lactate did not significantly decrease on the morning after admission. Whether this was indicative of an ongoing inflammatory process or adequacy of perfusion is difficult to determine. The role of microbial-derived endotoxin appears to play a minor role in our study: the clinical suspicion of infection during ICU stay was brought on only in a few subjects and even less had proven infection during the course of their ICU stay (Table 4). Moreover, the intraoperative hemodynamic variables were similar between EA stratification groups (Table 3). However, the prevalence of high endotoxin levels in patients who underwent thoracic surgery and the trend towards a relative hypoxemia despite more aggressive ventilatory management in patients with high EA levels is of interest. Both hypoxemia [24] and mechanical ventilation [25] are related to endotoxemia, even if we cannot exclude the potential higher prevalence of cigarette smokers in the thoracic group [26]. Except for obese patients that represent a unique topology, patients that underwent thoracic and abdominal procedures were similar to the others, with respect to age, APACHE score, and functional data at ICU admission. This suggests that measurement of EA is a potential tool to stratify patients to more aggressive care or to allocate resources in dynamic ICUs recruiting post-operative patients for routine monitoring. Whether EA stratification is useful only for abdominal and thoracic procedures cannot be determined from our data: the number of subjects does not allow for multivariate analysis. Moreover, we do not have EA data prior to surgery in order to discriminate between patients who presented to the operating room with pre-existing endotoxemia that might have persisted after the surgical procedure itself. These are interesting aspects that need further attention.

There are limitations to our study. Because of logistical reasons measurements were available only during the week days: this may have introduced a selection bias. As discussed above, pre-operative evidence of endotoxemia is lacking: this would have added to the interpretation of the data. Moreover, the number of patients recruited is not high enough to generalize our results to a wider ICU population.

## Conclusions

In this study we have investigated the prevalence of endotoxemia in a population of patients admitted to an ICU after surgery. A number of patients were characterized by high levels of endotoxemia, as assessed by EA assay, despite their low level of complexity on admission. High levels of endotoxin were associated with a longer ICU length of stay, particularly in patients who underwent major surgery.

## Key messages

• Endotoxemia is detectable in patients admitted to the ICU after surgery.

• High levels of endotoxemia are associated with longer ICU length of stay.

## Abbreviations

APACHE: Acute Physiology and Chronic Health Evaluation; EA: endotoxin activity; ICU: intensive care unit; LAL: Limulus-Amebocyte-Lysate; SIRS: Systemic Inflammatory Reaction Syndrome.

## Competing interests

The authors declare that they have no competing interests.

## Authors' contributions

FV conceived the study, collected and analysed the data, and wrote the manuscript. LF collected and analysed the data, and wrote the manuscript. SC, SF, FS, CT, MM, MP, and VS collected the data and performed analysis. MLR collected the microbiologic data. CM collected the data. LG wrote the manuscript.
